# Validation of death prediction after breast cancer relapses using joint models

**DOI:** 10.1186/s12874-015-0018-x

**Published:** 2015-04-01

**Authors:** Audrey Mauguen, Bernard Rachet, Simone Mathoulin-Pélissier, Gill M Lawrence, Sabine Siesling, Gaëtan MacGrogan, Alexandre Laurent, Virginie Rondeau

**Affiliations:** Biostatistic unit, INSERM U897, ISPED, Université de Bordeaux, 146 rue Léo Saignat, Bordeaux Cedex, 33076 France; Cancer Research UK Cancer Survival Group, Department of Non-Communicable Disease Epidemiology, London School of Hygiene and Tropical Medicine, Keppel Street, WC1E 7HT, London, UK; Clinical epidemiology and research, Institut Bergonié, 229 Cours de l’Argonne, Bordeaux, 33000 France; INSERM CIC-EC7, ISPED, Université de Bordeaux, 146 rue Léo Saignat, Bordeaux Cedex, 33076 France; West Midlands Cancer Intelligence Unit, 5, St Philip’s Place, Birmingham, B3 2PW UK; Comprehensive Cancer Centre The Netherlands (IKNL), Godebaldkwartier 419 ingang Janssoenborch, Utrecht, 3511 The Netherlands

**Keywords:** Breast cancer, Joint frailty model, Landmark, Prediction, Relapse history, Survival

## Abstract

**Background:**

Cancer relapses may be useful to predict the risk of death. To take into account relapse information, the Landmark approach is popular. As an alternative, we propose the joint frailty model for a recurrent event and a terminal event to derive dynamic predictions of the risk of death.

**Methods:**

The proposed prediction settings can account for relapse history or not. In this work, predictions developed on a French hospital series of patients with breast cancer are externally validated on UK and Netherlands registry data. The performances in terms of prediction error and calibration are compared to those from a Landmark Cox model.

**Results:**

The error of prediction was reduced when relapse information was taken into account. The prediction was well-calibrated, although it was developed and validated on very different populations. Joint modelling and Landmark approaches had similar performances.

**Conclusions:**

When predicting the risk of death, accounting for relapses led to better prediction performance. Joint modelling appeared to be suitable for such prediction. Performance was similar to the landmark Cox model, while directly quantifying the correlation between relapses and death.

**Electronic supplementary material:**

The online version of this article (doi:10.1186/s12874-015-0018-x) contains supplementary material, which is available to authorized users.

## Background

Individual predictions are increasingly sought after to help guide treatment decisions and patient care. Accurate predictions are especially important in the context of personalised medicine, where the ultimate goal is to give personalised targeted treatment to every patient. To do so, it is important to evaluate patient prognosis, accounting for their individual characteristics. In recent years, prognosis research in cancer has focused mainly on the presence of biomarkers that can be targeted by treatments. Less focus has been given to the impact of relapses that patients may experience, such as loco-regional relapses or distant metastases in cancer patients, which may explain a large part of the risk of death, despite adequate methods of analysis currently available. The impact of these relapses on the risk of death may vary according to the type of cancer. Relapses can be considered as a surrogate for patient frailty or for disease aggressiveness. It is therefore of interest to investigate how these events can be used to predict patient survival.

Relapses are recurrent events, evolving during patient follow-up. Thus it is of interest to study the impact of recurrences on death. However, relapses and death are non-independent events, sometimes called semi-competing risks, and relapses cannot be included in a survival model as a standard time-dependent covariate to study the risk of death [1]. The Landmark approach [2,3] resolves the problem by updating the population of interest. At each chosen prediction time, the model is estimated again on patients still alive. Thus, relapse history, observed before the prediction time, can be resumed as a baseline covariate, such as the number of previous relapses. This method offers the advantage of a simple and robust model. However, to do some dynamic predictions using several prediction times, several models have to be run on sub-populations of alive patients. Moreover, summarising the relapse process in a single variable may result in a loss of information.

Alternatively, joint modelling can be used to study recurrent events [4,5]. The hazard of the recurrent event and the hazard of the terminal event (death) are jointly modelled. Such models allow us to fully consider the correlation between the two processes using a shared random effect (frailty). Dynamic predictions can then be derived, accounting for all previous events. Once the parameters are estimated, predictions can be updated without running the model again. A recent work investigated the impact of relapses on the risk of death in breast cancer using a joint frailty model [6]. The proposed method was shown to be an adequate framework for prediction, and the model seemed to give a satisfying performance on the sample used to develop the model (development sample). However, the high number of parameters to be estimated may be a concern in prediction context, especially for generalisability purpose. It is therefore essential to validate the proposed prediction on independent data [7-9].

Here, our main goal is to validate a method of predictions from a joint model for recurrent events and death as accurate predictions. We present the results of the external validation of the previously developed prediction based on relapses in addition to well-known prognostic factors [6]. In order to assess whether the high number of parameters is a concern, we compare this new prediction performances with those of a Landmark Cox model. As in the development step, we apply the proposed prediction on breast cancer cases, here from two population-based registries, in West Midlands (England) and the Netherlands.

Section “Methods” of this paper explains the prediction probabilities within the framework of a Landmark Cox model and a joint frailty model, as well as the tools to validate them. The validation of the prediction on the West Midlands and Netherlands registry datasets is developed in section “Results”. Finally, sections “Discussion” and “Conclusion” contain concluding remarks.

## Methods

We are interested in the prediction of the risk of death between a prediction time *s* and a prediction horizon *s*+*w* considering all the information available at time *s*. The information includes some baseline covariates, but also history of recurrent events (loco-regional relapse or distant metastasis) until time *s*. In this context, the predicted risk of death can be updated after each new recurrence.

### Prediction of death in the joint modelling framework

The joint frailty model for a recurrent event and a terminal event is defined as follows [5]: for subject *i* (*i*=1,…,*N*), let *X*_*ij*_ be the *j*^*th*^ recurrent time (*j*=1,…,*n*_*i*_) measured from the study origin (calendar time), *D*_*i*_ be the death time and *C*_*i*_ be the independent censoring time. Note that the censoring time can happen to be a recurrence time. $T^{R}_{\textit {ij}}=min(X_{\textit {ij}},C_{i},D_{i})$ corresponds to each follow-up time and $\delta ^{R}_{\textit {ij}}$ is a binary indicator for recurrent events which is 0 if the observation is censored or if the subject died, and 1 if *X*_*ij*_ is observed $\left (\delta ^{R}_{\textit {ij}}\right.=I\left [T^{R}_{\textit {ij}}=X_{\textit {ij}}\right ]$ where *I*[.] denotes indicator function). Similarly, we note ${T^{D}_{i}}$ as the last follow-up time for subject *i*, which is either a time of censoring or a time of death $\left ({T^{D}_{i}}=min(C_{i},D_{i})\right)$ and ${\delta ^{D}_{i}}=I\left [{T^{D}_{i}}=D_{i}\right ]$. We actually observe the sequence $\left (T^{R}_{\textit {ij}},\delta ^{R}_{\textit {ij}},{T^{D}_{i}},{\delta ^{D}_{i}}\right)$. Finally, we denote by $Z^{R}_{\textit {ij}}$ and ${Z^{D}_{i}}$ the vectors of covariates associated with the hazard of recurrent events and death, respectively. However, a patient is considered at risk of a *j*^*th*^ recurrence only after the (*j*−1)^*st*^ recurrence. The joint model is then written as:

(1) $$\begin{array}{@{}rcl@{}}  \left\lbrace \begin{array}{l} \lambda^{R}_{ij}(t|u_{i}) =u_{i}{\lambda^{R}_{0}}(t)\exp\left(\beta_{1}'Z^{R}_{ij}\right) =u_{i} \lambda^{R}_{ij}(t)\\ {\lambda^{D}_{i}}(t|u_{i}) =u_{i}^{\alpha}{\lambda^{D}_{0}}(t)\exp\left(\beta_{2}'{Z^{D}_{i}}\right)=u_{i}^{\alpha} {\lambda^{D}_{i}}(t) \end{array} \right. \end{array} $$

where ${\lambda ^{R}_{0}}(.)$ is the baseline hazard of a recurrent event, irrespective of event rank, and ${\lambda ^{D}_{0}}(.)$ the baseline hazard of death. The effects of explanatory variables *β*_1_ and *β*_2_ are assumed to be different for the hazard of recurrent events and the risk of death. The two processes are linked by the patient-specific frailty effect *u*_*i*_. The frailty terms are independent and identically distributed following a gamma distribution with variance *θ* and, without loss of generality, a mean equal to 1. That is:

(2) $$ {\small{\begin{aligned} u_{i} \sim \text{Gamma}\left(\frac{1}{\theta};\frac{1}{\theta}\right) \quad \text{and} \quad g(u_{i})=\frac{u^{1/\theta-1}\exp(-u/\theta)}{\theta^{1/\theta} \Gamma(1/\theta)} \end{aligned}}}  $$

The baseline hazard functions (${\lambda ^{R}_{0}}(.)$ for recurrent events and ${\lambda ^{D}_{0}}(.)$ for death) are approximated using cubic splines, but alternative flexible functions such as fractional polynomials could be used. Splines are piecewise polynomials that are constrained to smoothly joint to fit curves. We used ${\lambda ^{R}_{0}}(.) = \sum _{i=1}^{m} {\eta ^{R}_{i}} M_{i}(.)$ and ${\lambda ^{D}_{0}}(.) = \sum _{i=1}^{m} {\eta ^{D}_{i}} M_{i}(.)$ where *M*_*i*_(.) is the common basis splines, and *η*^*R*^ and *η*^*D*^ the two vectors of splines coefficients. The parameters of the model *ξ*=(*η*^*R*^,*η*^*D*^,*β*_1_,*β*_2_,*α*,*θ*) are estimated using penalized maximum likelihood estimators. For more details on the inference method, please see [5]. To estimate this model, all the available information is used, from the origin to the end of follow-up of all patients.

Using the joint modelling framework, we are interested in two prediction settings previously defined [6]. The first prediction of the risk of death *P*^*Rec*^ is calculated based on all relapses information. In this setting, the *J* relapses occurring before the prediction time *s* are considered (*J*≤*n*_*i*_). We consider the patient history $\mathcal {H}_{i}^{J}(s)=\{{N_{i}^{R}}(s)=J, X_{i1}<\ldots <X_{\textit {iJ}} \le s \}$, with *X*_*i*0_=0 and *X*_*i*(*J*+1)_>*s*, to define the conditional probability of death *P*^*Rec*^ as follows:

(3) $${} {\footnotesize{\begin{aligned} &P^{Rec}(s,s+w;\xi) \\ &\,=\, P\left(\!D_{i}\le s+w|D_{i}>s, \mathcal{H}_{i}^{J}(s), Z^{R}_{s,ij}, Z^{D}_{s,i},\xi\!\right)\\ &\,=\, \frac{\int_{0}^{\infty} \left[{S^{D}_{i}}\left(s|Z^{D}_{s,i}, u_{i},\xi\right)\,-\,{S^{D}_{i}}\left(s+w|Z^{D}_{s,i}, u_{i}, \xi\right)\!\right] (u_{i})^{J} S^{R}_{i(J+1)}\!\left(\!s|Z^{R}_{s,ij}, u_{i}, \xi\!\right)\! g(u_{i}) \mathrm{d}u_{i}} {\int_{0}^{\infty} {S^{D}_{i}}\left(s|Z^{D}_{s,i}, u_{i},\xi\right) (u_{i})^{J} S^{R}_{i(J+1)}\left(\!s|Z^{R}_{s,ij}, u_{i},\xi\!\right) g(u_{i}) \mathrm{d}u_{i}} \end{aligned}}}  $$

where $Z^{R}_{s,ij}$ and $Z^{D}_{s,i}$ are the values of the covariates at time *s*, and *g*(*u*_*i*_) is the density of the gamma distribution defined in equation (2).

The second setting *P*^*Ign*^ also uses the joint modelling framework. However, while recurrences information is used in model estimation, the information about previous recurrences is not considered in the prediction, and it can be missing. It is defined by:

(4) $$ {\small{\begin{aligned} &P^{Ign}(s,s+w;\xi) \\ &= P\left(D_{i}\le s+w|D_{i}> s, Z^{D}_{s,i},\xi\right) \\ & = \frac{\int_{0}^{\infty} \left[{S^{D}_{i}}\left(s|Z^{D}_{s,i}, u_{i},\xi\right)-{S^{D}_{i}}\left(s+w|Z^{D}_{s,i}, u_{i},\xi\right)\right] g(u_{i})\mathrm{d}u_{i}} {\int_{0}^{\infty} {S^{D}_{i}}\left(s|Z^{D}_{s,i}, u_{i},\xi\right) g(u_{i}) \mathrm{d}u_{i}} \end{aligned}}}  $$

Both settings are dynamic in the sense that the prediction can be updated by changing the prediction time *s*, thus the quantity of available information, and/or the prediction window *w*. The first setting considers the individual relapse history, whereas the second ignores it.

### Prediction of death using a Landmark Cox model

The Landmark approach involves fixing a prediction time *s* and fitting the model on the sub-group of patients still at risk of death at this time, that is, patients alive and not lost to follow-up [2]. Thus, the number of relapses occurring before time *s* can be treated as a baseline covariate, and the recurrences occurring after *s* are ignored. This covariate can be updated when another Landmark time *s* is considered, and a new model is fitted. With this approach, a robust model can be used, requiring few parameters, and the time-dependent effects are easily dealt with. However, only a sub-group of alive patients is included to fit the model, which may result in a loss of information in the parameter estimation.

We have *D*_*i*_ the death time and *C*_*i*_ the independent censoring time. Let $ \lambda ^{D}_{s,i}\left (.|Z^{D}_{s,i}\right)$ denote the hazard function of death conditional on being alive at the Landmark time *s*, $\lambda ^{D}_{s,0}(.)$ be the conditional baseline hazard function, $Z^{D}_{s,i}$ be the covariate vector at time *s* and *β*_*s*_ be their effect estimated at time *s*. The Landmark Cox model is then written as follows:

(5) $$ \lambda^{D}_{s,i}\left(t|Z^{D}_{s,i}\right) = \lambda^{D}_{s,0}(t) \exp\left(\beta_{s}'Z^{D}_{s,i}\right), \text{for}\, t \geq s  $$

This model is estimated with the information available at the Landmark time *s*. The prognostic factors of interest $Z^{D}_{s,i}$ may include information about previous recurrent events, for example, their frequency and timing. The baseline hazard of death $\lambda ^{D}_{s,0}(.)$ is estimated using splines with parameters ${\eta ^{D}_{s}}$. The parameters of the model $\xi ^{LM}_{s} = ({\eta ^{D}_{s}},\beta _{s})$ are estimated using penalized maximum likelihood estimators as in [5]. The corresponding prediction of the risk of death is written as:

(6) $$ \begin{aligned}  &P^{LM}\left(s,s+w;\xi^{LM}_{s}\right) \\ &= P\left(D_{i}\le s+w|D_{i}> s, Z^{D}_{s,i},\xi^{LM}_{s}\right) \\ &= \frac{{S^{D}_{i}}\left(s|Z^{D}_{s,i}, \xi^{LM}_{s}\right)-{S^{D}_{i}}\left(s+w|Z^{D}_{s,i}, \xi^{LM}_{s}\right)}{{S^{D}_{i}}\left(s|Z^{D}_{s,i}, \xi^{LM}_{s}\right)} \end{aligned}  $$

where ${S^{D}_{i}}(.|Z^{D}_{s,i}, \xi ^{LM}_{s})$ is the survival function conditional on being alive at time *s* associated to the hazard of death.

### External validation of the prediction

In order to make predictions using the three proposed settings *P*^*LM*^, *P*^*Rec*^ and *P*^*Ign*^ on new patients, the model parameters are estimated on the development sample. Based on these estimators, predictions for new patients are obtained by replacing the patient level information, $Z^{D}_{s,i}$ in equation (6) or *J*, $Z^{R}_{s,ij}$ and $Z^{D}_{s,i}$ in equations (3) and (4), with actual information on the new patient.

The quality of fit of the two models can be compared on the development data using an approximate likelihood cross-validation criterion as in [10].

#### Prediction error

To estimate if the predictions are accurate, error of prediction curves are used, based on the Brier score. The Brier score aims to measure how far the prediction is from the actual outcome of the patients. We used a weighted estimator of the Brier score to account for right censoring using the Inverse Probability of Censoring Weights (IPCW) [11].

Let *N*_*s*_ be the number of patients alive and uncensored at prediction time *s*, that is, patients for whom the prediction can be made. Given ${T^{D}_{i}}$ the possibly right-censored survival time, ${\delta ^{D}_{i}}$ the corresponding event indicator (${\delta ^{D}_{i}}=1$ if the observed time is a death time, 0 otherwise). We denote $\widehat {G}_{N}(.)$ the Kaplan-Meier estimate of the censoring distribution on the sample. Using the generic term $\hat {P}(s,s+w;\hat {\xi })$ which can be one of the three prediction probability settings previously described, the error of prediction is defined by:

$${\small{\begin{aligned} Err_{s,w} &= \frac{1}{N_{s}} \sum\limits_{i=1}^{N_{s}} \left[I\left({T^{D}_{i}}>s+w\right)-\left(1- \hat{P}\left(s,s+w;\hat{\xi}\right)\right)\right]^{2}\\ &\quad\times\hat{h}_{i}\left(s+w,\hat{G}_{N}\left(.\right)\right) \end{aligned}}} $$

with $\hat {h}_{i}(s+w,\hat {G}_{N}(.))$ being a weight that accounts for right censoring:

$${} \hat{h}_{i}\left(s+w,\hat{G}_{N}\left(.\right)\right) = \frac{I\left({T^{D}_{i}} \leq s+w\right){\delta^{D}_{i}}}{\hat{G}_{N}\left({T^{D}_{i}}\right)/\hat{G}_{N}\left(s\right)} + \frac{I\left({T^{D}_{i}} > s+w\right)}{\hat{G}_{N}\left(s+w\right)/\hat{G}_{N}\left(s\right)} $$

The performance of the models is compared using a measure of explained residual variation defined as follows [12]:

$$R^{2} = 1 - \frac{Err_{s,w}}{Err_{s,w;KM}} $$

where *Err*_*s*,*w*_ is the error of one of the predictions (*P*^*LM*^, *P*^*Rec*^ or *P*^*Ign*^) described as above and *Err*_*s*,*w*;*KM*_ is the error of prediction using the Kaplan-Meier estimate at *s*+*w* in the entire set of patients. It can be interpreted as how much the prediction error is decreased using the model-based prediction as compared to an average prediction estimated by Kaplan-Meier.

The proposed error of prediction is calculated in two different ways: either *s* is fixed and *w* varies, or *s* varies while *w* is fixed.

#### Calibration plot

Another indicator of the accuracy of the prediction tool proposed is the calibration [13]. A well-calibrated prediction means that, among 100 patients with a predicted event risk of *p%*, *p* of them will actually experience the event. This can be computed only for a binary endpoint, meaning that we must choose a time of prediction. We set it at *s*+*w*=10 years.

The calibration is illustrated using a calibration plot. The predicted risks of death are grouped according to the deciles of their distribution. For each decile, the observed proportion of an event is plotted against the mean predicted value, along with the 95*%* confidence interval for the observed proportion. For a well-calibrated prediction, all points should fall very close to the firstbisector.

On the calibration plot the histogram of the predicted values is also represented, showing how they are distributed between 0 and 1.

#### Software

Joint model and predictions were computed using the R functions *frailtyPenal* and *prediction* from the R package *frailtypack* [14]. The Brier score were computed using the R package *pec* [15]. The calibration plots were drawn using R software (code available as Additional file 1).

#### Ethical approval and availability of supporting data

For the French hospital series, ethical approval from the national ethics committee (Commission Nationale de l’Informatique et des Libertés) was obtained for this study, which allowed the use of data recorded in this clinical and pathological database. In this comprehensive cancer center, each patient was informed that medical data can be use in observational research. The procedure follows the French law for medical research. We hold statutory and ethical approvals to analyse the data from West Midlands Cancer Intelligence Unit and Comprehensive Cancer Centre The Netherlands registry data.

All data used are confidential. Researchers may access the data by sending a formal request to the appropriate institution (Institut Bergonié for the French series, West Midlands Cancer Intelligence Unit for the UK registry data and Comprehensive Cancer Centre The Netherlands for the Dutch registry data).

## Results

### Population comparison

The first validation sample consisted of all breast cancer cases diagnosed in West Midlands, England, in 1996 and followed until 2012. The second validation sample consisted of cases from the Netherlands Cancer Registry, South Netherlands region excluded, diagnosed between 2003 and 2006 and followed until the end of 2012. Because the vital status of the patients is ascertained in both registries through a passive approach, no lost to follow-up is assumed. The development cohort consisted of 1067 patients operated in a comprehensive cancer centre between 1989 and 1993, and with a median follow-up of 14 years. Thus, the two validation populations differ from the development population in terms of country and selection of population (general population in West Midlands and Netherlands; hospital-based patients in France) and inclusion period (1996 and 2003-2006 vs. 1989-1993).

In the two validation samples, a high rate of missing data was observed. Out of the 3168 cases recorded in the year 1996 in West Midlands, 1196 (38%) had non-missing values for all of the five studied prognostic factors. In the Dutch sample, information about peritumoural vascular invasion was not recorded. Of the 41,676 recorded patients, 31,075 (75%) had non-missing values for the four remaining factors. In our validation sample, we included only patients with complete information in the two datasets. This decision is discussed in the last part of the paper.

Table 1 compares the repartition of the prognosis factors in the three samples, as well as the number of relapses per patient, and the overall survival. Patients in both validation samples had more severe disease, i.e., more peritumoural vascular involvement (38.5% in West Midlands vs. 26.7%), a tumour size greater than 20 mm (46.8% in West Midlands and 39.8% in Netherlands vs. 22.7%) and grade III disease (37.1% in West Midlands and 33.8% in Netherlands vs. 24.6%), despite a similar age. As a result, overall survival in both West Midlands and Netherlands was lower than in the developmentcohort.

**Table 1:** **Description of the three samples used to develop (n =1067) and validate the model (n =3168 and n =31,075)**

	**French cohort**		**West Midlands**		**Netherlands**	
	**(1989-1993)**		**(1996)**		**(2003-2006)**	
**Variable**	**N=1067**	**%**	**N=1196**	**%**	**N=31075**	**%**
Age						
Age ≤40	82	7.7	73	6.1	2126	6.8
Age [40-55]	391	36.6	456	38.1	10681	34.4
Age >55	594	55.7	667	55.8	18268	58.8
Peritumoural vascular involvement	285	26.7	460	38.5	-	-
Tumour size > 20 mm	242	22.7	560	46.8	12365	39.8
Nodal involvement	451	42.3	496	41.5	12588	40.5
Grade						
Grade I	316	29.6	226	18.9	6565	21.1
Grade II	488	45.7	526	44.0	13993	45.0
Grade III	263	24.6	444	37.1	10517	33.8
Number of recurrent events						
None	705		895		27231	
1	301		240		3834	
2	57		49		10	
3	4		10		0	
4	0		2		0	
5-year survival	89.1	(87.3-91.0)	76.6	(74.2-79.0)	85.5	(85.1-85.9)
10-year survival	77.1	(74.6-79.7)	63.1	(60.5-65.9)	-	
15-year survival	65.4	(62.2-68.2)	51.6	(48.8-54.5)	-	

The number of relapses per patient also varied. There were up to four relapses recorded in the West Midlands sample compared with a maximum of two in the Dutch sample and three in the development cohort. In the West Midlands registry, relapses were not collected but retrieved from the treatment information with an algorithm that uses the treatment type and time interval between successive treatments [16]. In the Dutch sample, relapse data was obtained directly from patient files; both clinically and pathologically confirmed relapses were recorded. The recording was limited to relapses occurring during the first five years after diagnosis and, in some regions, to the first relapse of each type (local relapse, regional relapse or distant metastasis). In the French cohort, relapses (loco-regional recurrence or distant metastasis) were recorded following a clinical examination. That resulted in 75% of the patients without registered relapse in the West Midlands, 88% in the Dutch sample, and 66% in the French cohort.

### Validation of the prediction

#### Models

The results of the joint frailty model and the Landmark Cox model (thereafter called Landmark model), estimated on the French data, are shown in Table 2. The prognostic factors kept for prediction were those associated with the risk of recurrent events or with the risk of death in the joint model. The joint model estimation (first and second columns) showed that the risk of recurrent event was higher in younger women (age ≤40), in women with peritumoural vascular involvement, in women with larger tumour (tumour size >20 mm), in women with nodal involvement, and in women with grade other than I. The risk of death was decreased for women between 40 and 55-year old, and increased in women with peritumoural vascular involvement, with large tumour size, with nodal involvement, and with grade other than I. The variance of the random effects *θ* differed significantly from zero, meaning that there exists an heterogeneity between patients regarding their risk of recurrence and death, and that this heterogeneity could not be totally explained by the measured prognostic factors. The *α*>0 showed that the patients at higher risk of relapses were also at higher risk of death. Results of the Landmark model are shown in the last column. The main difference is that only the effect of covariates on the risk of death are estimated, and the estimations were adjusted parametrically on the number of relapses. As a consequence, the Landmark model provided lower estimated effects than with the joint model. After adjustment on the number of relapses, only the presence of a nodal involvement was significantly associated with a higher risk of death, and both age lower than or equal to 40, and age between 40 and 55 years were associated with a lower risk of death. The Landmark model also showed an important effect of the number of previous relapses. The likelihood cross-validation criterion was lower for the Landmark model (0.93 *versus* 1.19), suggesting that this model fitted the data better than the joint model. However, a better fit does not necessarily result in better prediction [17].

**Table 2:** **Joint and Landmark Cox models estimations on the French cohort (n=1067 patients, 427 recurrent events)**

	**Joint model**				**Cox landmark**	
	**For recurrent events**		**For death**		**For death**	
**Variable**	**HR**	**(95% CI)**	**HR**	**(95% CI)**	**HR**	**(95% CI)**
Age						
[40−55] vs. >55	1.17	(0.91-1.51)	0.31	(0.16-0.60)	0.56	(0.41-0.76)
≤40 vs. >55	2.41	(1.73-3.37)	1.57	(0.73-3.38)	0.54	(0.31-0.92)
Peritumoural vascular involvement	1.61	(1.26-2.06)	4.74	(2.54-8.85)	1.04	(0.76-1.43)
Tumour size (>20 mm vs. ≤20 mm)	1.95	(1.52-2.50)	6.21	(2.99-12.86)	1.20	(0.88-1.65)
Nodal involvement	1.84	(1.44-2.36)	4.89	(2.47-9.67)	1.95	(1.45-2.60)
Grade						
*II vs. I*	2.18	(1.57-3.01)	7.48	(2.71-20.66)	1.07	(0.75-1.52)
*III vs. I*	3.09	(2.16-4.41)	44.33	(15.61-125.93)	1.25	(0.83-1.88)
Recurrences before *t*=5 years						
One previous recurrence					7.18	(5.25-9.83)
Two previous recurrences					6.94	(3.05-15.83)
*θ*=*v* *a* *r*(*u*_i_)	1.07	(se=0.06)				
*α*	4.45	(se=0.33)				
LCV	1.19				0.93	

HR: Hazard ratio; CI: Confidence interval; LCV: Likelihood cross-validation criterion; Cox Landmark at time t = 5 years.

#### Prediction error

**Figure 1:**
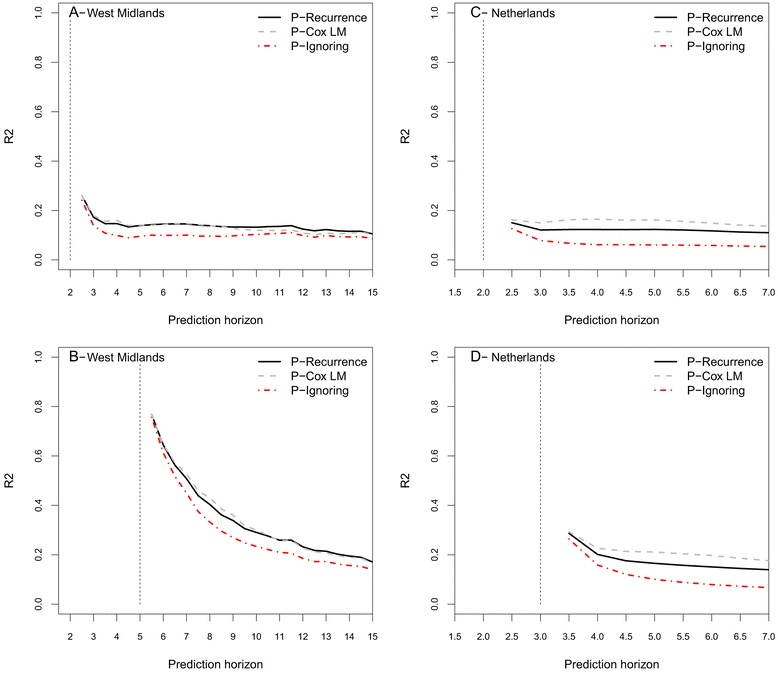
**Error of prediction on validation samples for the three predictions.** **A**. On the West Midlands sample, at the prediction time *t*=2 years and a prediction horizon from 2.5 to 15 years. **B**. On the West Midlands sample, at the prediction time *t*=5 years and a prediction horizon from 5.5 to 15 years. **C**. On the Dutch sample, at the prediction time *t*=2 years and a prediction horizon from 2.5 to 7 years. **D**. On the Dutch sample, at the prediction time *t*=3 years and a prediction horizon from 2.5 to 7 years.

Overall, all of the studied prediction settings gave better results than the Kaplan-Meier, with a higher *R*^2^ for both predictions accounting for relapses (Figure 1). In the West Midlands, when the time of prediction *s* is at five years (Figure 1B), *R*^2^ was as high as 80% for early predictions and regularly decreased with increasing prediction horizon (30% at 10 years). The gain in the prediction error diminished with the prediction horizon, being around 50% at seven years and ending around 17% at 15 years, showing that the information from the model had a higher impact on short-term prediction. *R*^2^ was still around 20% at a horizon of 15 years, but very similar for the three settings. *R*^2^ was low, under 20%, for shorter prediction time (*s*=2 years; see Figure 1A). This illustrates the fact that the information gathered up to two years was not enough to obtain good prediction, especially considering relapses. In the Dutch sample (Figures 1C and D), the limited follow-up prevented us from studying prediction times longer than three years. The results were very similar to those in the West Midlands sample at *s*=2 years, but the difference between the three settings was larger. Results were better for the prediction from the Landmark model, and worse for the prediction ignoring the relapse information.

**Figure 2:**
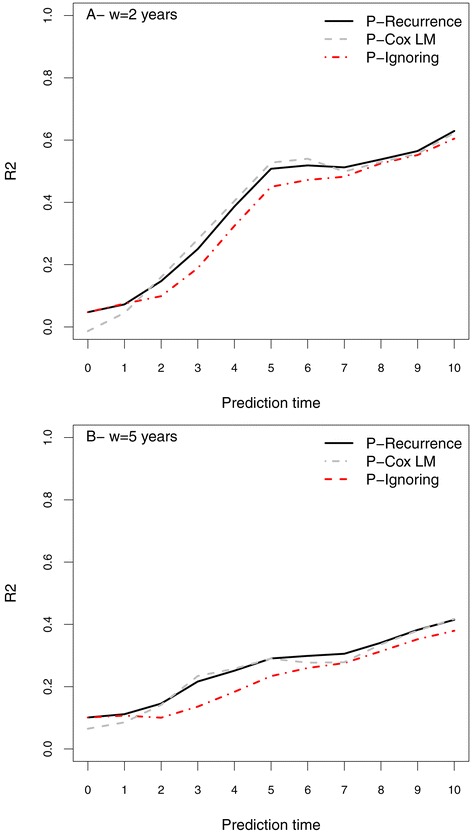
**Error of prediction on the West Midlands sample when the prediction time** ***t*** **is increasing from 0 to 10 and the window of prediction is set at A.** 2 years and **B**. 5 years.

When holding the prediction window at *w*=2 or *w*=5 years, results were similar (Figures 2A and B, respectively). The setting ignoring relapses always gave lower performance, while the performances of the two other settings were very similar. The more information that was collected and used, the more accurate the prediction was, as shown by the curves increasing with time of prediction *t*, for both window times. As expected, the entire curve was higher (i.e., lower error of prediction) when the prediction was made for a shorter window (two years as compared to five years).

#### Calibration

**Figure 3:**
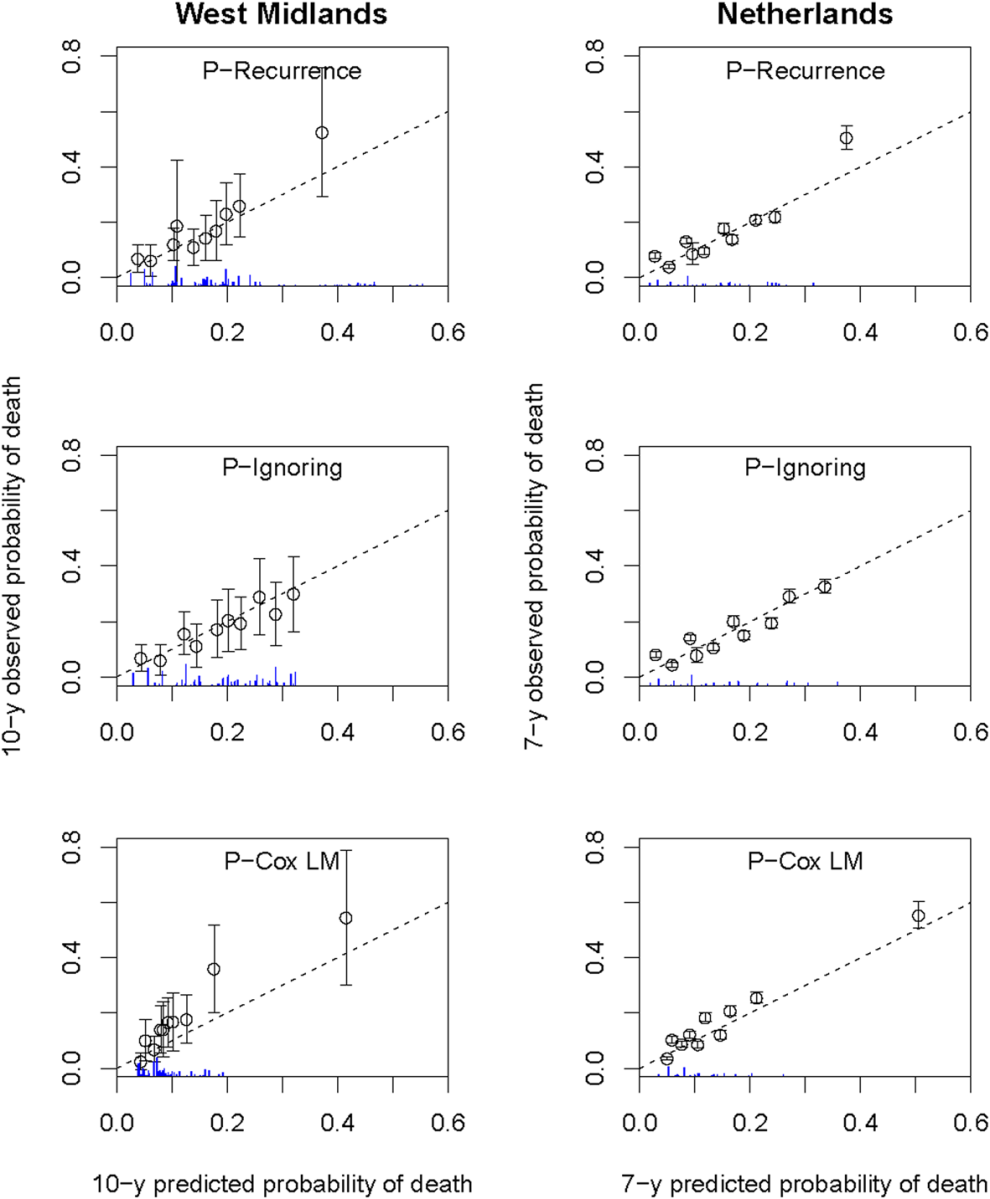
**Calibration plot for the three predictions of death between 5 and 10 years in the West Midlands sample (left panel) and between 2 and 7 years in the Dutch sample (right panel).**

All three settings gave good calibration, with points around the first bisector (Figure 3). Interestingly, both prediction approaches accounting for relapses identified a group of patients with high risk of death in both samples. For these high-risk patients, the mean predicted risk was somewhat lower than the observed risk (40% in the West Midlands vs. 50% in the Netherlands using *P*^*Rec*^). The histograms show that predicted values were lower overall for the Landmark approach (rarely exceeding 20%) whereas both predictions from the joint model gave higher risks. This may explain why the observed probability of death seemed underestimated with the Landmark approach in the West Midlands.

#### Additional validation by subgroups

The validation samples differed in many aspects from the development sample. Thus, even if good results were observed for the proposed prediction both in terms of prediction error and of calibration, it is crucial to check the accuracy of the prediction on a more similar sample. For these subgroups analyses, the model is still developed on the whole French cohort; only the prediction is made on a subgroup of the English sample. Here we selected a subsample of operated patients, as it was the main selection criterion in the development sample.

**Figure 4:**
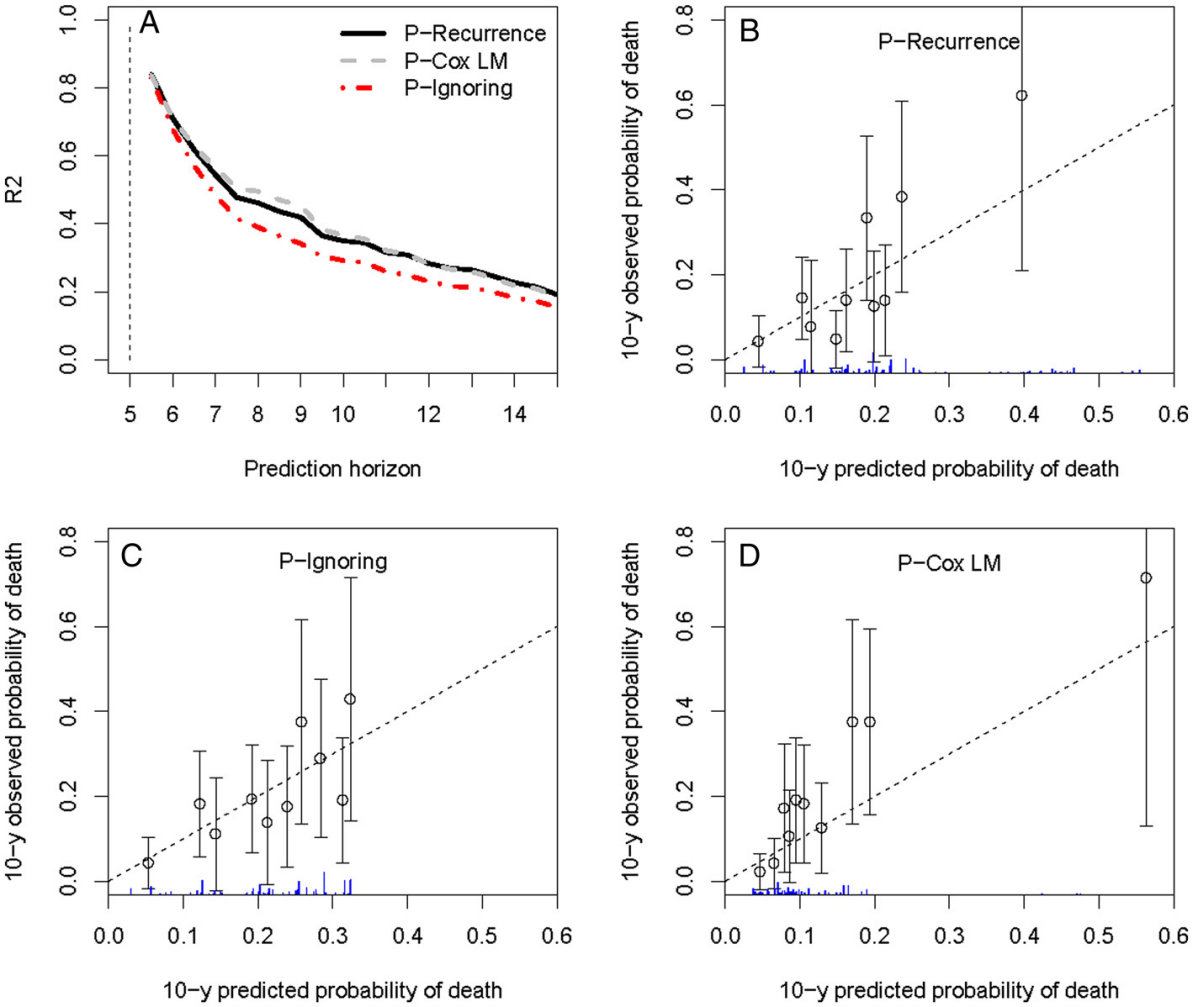
**Results of the prediction on the operated patients from West Midlands.** **A**. Relative prediction error at the prediction time *t*=5 years and a prediction horizon from 5.5 to 15 years. **B**,**C**,**D**. Calibration plots for the three predictions.

Similar results were observed with the 602 operated patients from the West Midlands (Figure 4). Large confidence intervals were observed due to the reduced number of subjects included in this analysis (n=417 patients alive at five years). Calibration was not better than as observed on the entire West Midlands data.

**Figure 5:**
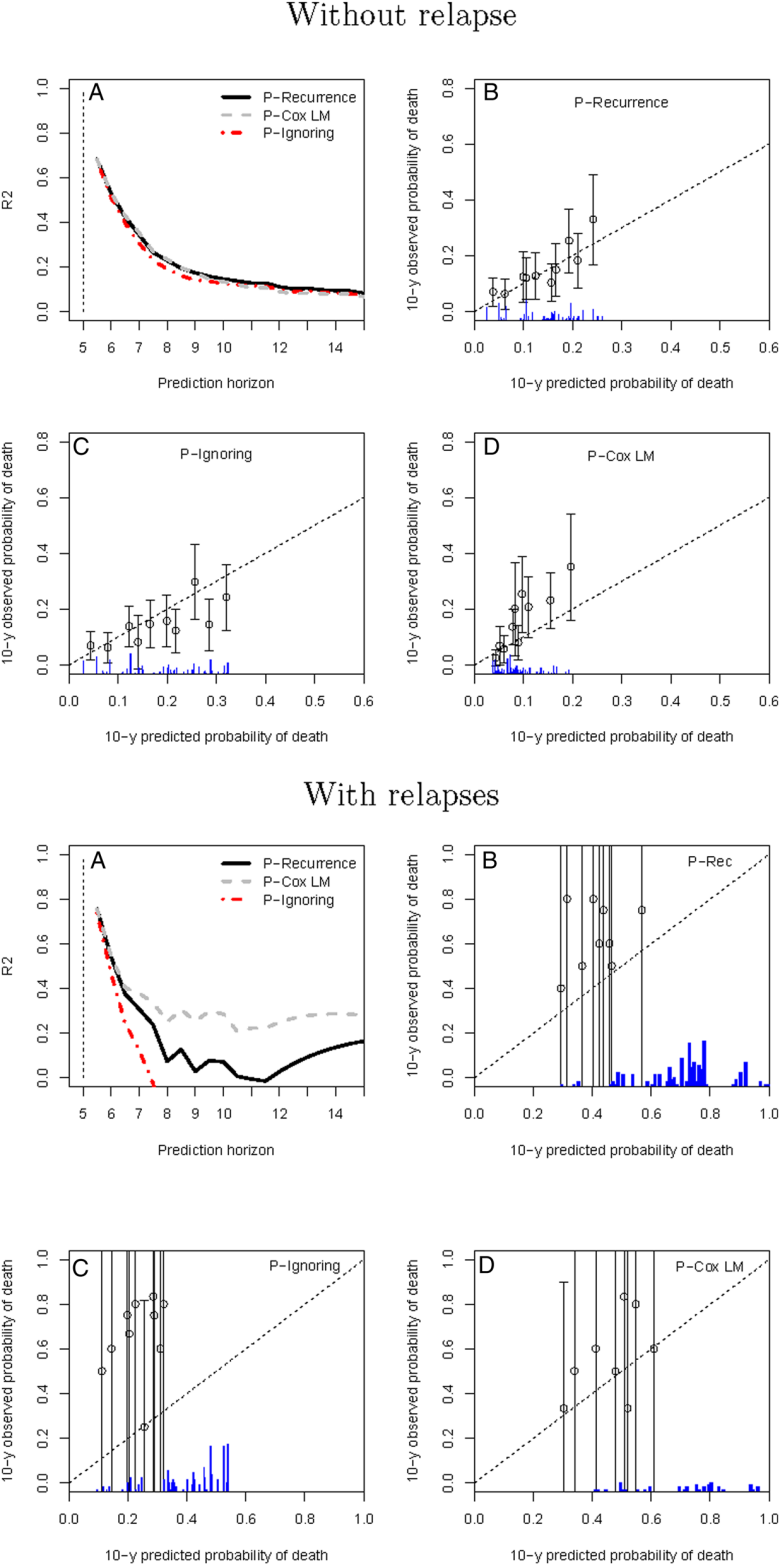
**Results of the prediction on the patients from West Midlands.** **A**. Relative prediction error at the prediction time *t*=5 years and a prediction horizon from 5.5 to 15 years. **B**,**C**,**D**. Calibration plots for the three predictions. The upper part is for patients without relapse before 5 years; the lower part is for patients with at least one relapse observed before 5 years.

A second subgroup analysis was performed comparing the performance of the proposed predictions between subjects who relapsed at least once before the prediction time of five years and those who did not (Figure 5). As expected, in the subgroup of patients without relapse, the results were very similar to those in the entire sample. However, no high-risk subject groups were identified, as observed in the main analysis. In the subsample with relapses, the prediction ignoring the relapses underestimated the observed probabilities of events (all the points are above the line) and had a very low *R*^2^, negative after 7.5 years.

## Discussion

To account for the recurrent events and their association with the risk of death, we used a joint frailty model for recurrent events and a terminal event. This framework appears suitable to derive such predictions. The obtained prediction gave a similar error of prediction to a Cox model in a Landmark approach. The two approaches have modelling assumptions. Both are proportional hazards models. In both models, we estimated the baseline hazards with a semi-parametric approach using splines. It was compared to a non-parametric estimation of the baseline hazard, and both approaches gave very similar results (data not shown). The landmarking approach allows the use of a simple and robust model, such as the Cox model. Moreover the covariates effects are re-estimated at each prediction times. However, only the alive patients are kept to estimate the model. By contrast, the joint model use all the sample patients and parameters are estimated once to do predictions. However, assumptions are made on the frailty structure and distribution. We can note that in the hypothesis of no association between the relapse and death processes (i.e., parameter *α*=0), the prediction of death in the joint model simplifies to an expression similar to equation (6), independent of the risk of relapses. Waiting for a more careful comparison of the two approaches, the choice between the joint and the Landmark approach should thus be mainly guided by the willingness (i) to fully describe both processes (recurrent event and death) and their correlation (joint model); or (ii) to focus only on the death (Landmark approach). A Landmark approach using a non-parametric prediction was also recently proposed to predict a long-term outcome accounting for a short-term event [18].

Initially, the proposed prediction incorporated information about the human epidermal growth factor receptor 2 (HER-2) status and hormonal receptor status. However, considering the non-availability of this information at the general population level at the time of this data collection, we have re-estimated the model and prediction without this information. On the initial development sample, we compared the prediction error of the two joint models, with and without this biological information. The prediction error was very similar for both models (data not shown).

One perspective of such prediction can be its use in clinical trials context. Indeed, the validation of surrogate markers in several cancers, such as the progression-free survival as a surrogate for the overall survival, raises the question about how to use the progression-free survival information in practice. One of the options is to use progressions to predict the risk of death in the two arms to be able to conclude earlier on the treatment effect [19]. In that perspective, the prediction that we validated maybe useful.

In the context of the joint modelling framework, the prediction of recurrent events can also be derived. Moreover, our analyses indicate that each prognostic factor considered separately adds very little prediction information once the baseline hazard and recurrent event processes are adequately modelled (data not shown). The covariates may be of greater interest when predicting the risk of a recurrent event. Considering relapse type differently (loco-regional relapse and metastasis) can also be of interest as they reflect various severity levels of the disease [20]. Finally, these predictions could be extended in the context of competing risks or excess mortality, where it would be possible to focus on predicting only the risk of death from cancer.

Finally, prognostic research literature is lacking when it comes to the consequences of missing data on the validation process, i.e., after the development phase. No prediction can be done if one of the covariates is missing. However, the impact of such exclusion on the validation process remains unclear. Multiple imputation has proved to be a useful approach for model estimation (e.g., [21]), and could also be used for the validation stage. However, the benefit of such imputation to estimate model performance is uncertain. To reproduce the conditions of clinical practice, we keep in our validation only the patients with complete information. The subjects with missing data were more likely to be 55 years and older, and to have more nodal involvement, for similar stage and tumour size (data not shown). As the predictions that take into account relapse information were demonstrated to be more appropriate in predicting high risk of death, it is possible that the performances of the prediction accounting for the relapses were underestimated. In the end, the survival results of the analysed patients were in accordance with the results of the EUROCARE-4 study [22]. In this study, the age-adjusted 5-year survival was 81% for French patients diagnosed between 1990 and 1994, and 78% and 83% for the patients diagnosed between 1995 and 1999 in England and Netherlands, respectively.

## Conclusion

The present work shows how recurrent events occurring in breast cancer patients may be used to obtain accurate prediction of death. The resulting calibration and error of prediction show that the estimated prognostic model is useful to predict the risk of death, in particular when enough variability in the number of recurrences is observed. Good calibration was obtained, especially considering that the validation samples differed from the development sample with respect to inclusion criteria for the patients and period, country and therefore, health care system. Using different incidence years is of great interest, since the care (especially treatment and screening) of breast cancer patients evolved during the 1990s, affecting survival. It seems that finally, despite these differences, the effect of covariates and relapses remained similar and was still of interest. Therefore, the joint model for recurrent event and a terminal event gives some accurate predictions, and the specific studied model in breast cancer can be used in different populations.

## Additional file

Additional file 1
**R code for drawing calibration plot.**
